# Supplementary material for: A spatially anchored transcriptomic atlas of the human kidney papilla identifies significant immune injury in patients with stone disease
Source: Nat Commun. 2023 Jul 19;14:4140. doi: 10.1038/s41467-023-38975-8 (PMC10356953; doi:10.1038/s41467-023-38975-8)
Supplement: Supplementary file 1 — Supplementary Information [file 41467_2023_38975_MOESM1_ESM.pdf]

SUPPLEMENTARY MATERIALS

MANUSCRIPT TITLE:

A spatially anchored transcriptomic atlas of the human kidney papilla identifies significant immune injury in patients with stone disease

AUTHORS

Victor Hugo Canela, William S. Bowen, Ricardo Melo Ferreira, Farooq Syed, James E. Lingeman, Angela R. Sabo, Daria Barwinska, Seth Winfree, Blue Lake, Ying-Hua Cheng, Joseph P. Gaut, Kaice A. LaFavers, Kun Zhang, Fredric L. Coe, Elaine Worcester, Sanjay Jain, Michael T. Eadon, James C. Williams, Jr., and Tarek M. El-Achkar, the Kidney Precision Medicine Project

Supplemental Figures

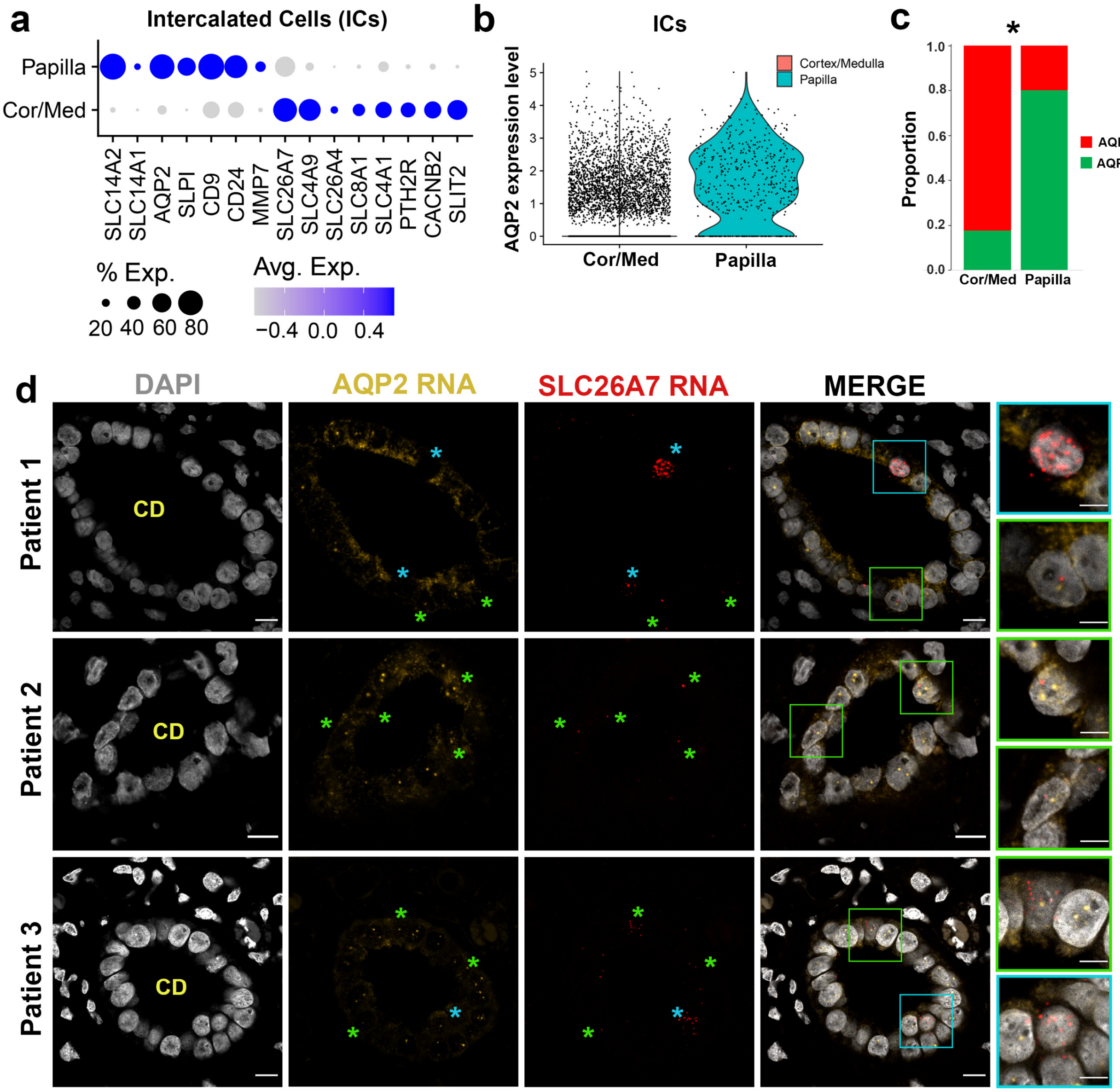

Supplementary Fig. 1: Intercalated cells in the papilla. (a) Differential gene expression between cortico-medullary and papillary intercalated cells based on snRNAseq (full dataset in Figure 1). Papillary intercalated cells have higher expression of AQP2 (b) than cortico-medullary ICs, and most IC cells in the papilla express AQP2 (c). Asterisk in (c) denotes statistical significance ($p < 0.0001$) using two tailed Fisher's exact test comparing proportions of AQP2 negative cells in the corticomedullary vs. papillary ICs. Panels in (d) show single molecular fluorescence in situ hybridization (smFISH) for AQP2 and SLC26A7 from 3 different stone papillary biopsy patients (scale bar = 10 μm). SLC26A7 is a chloride/bicarbonate exchanger specific to IC[1] and a marker for human IC cells from the atlas by Lake et al.[2] In the papilla, few IC collecting ducts cells express high levels of SLC26A7 with no AQP2 expression (cells

marked by blue asterisks). Many scattered collected duct cells express moderate SLC26A7 along with AQP2 (cells marked by green asterisks). The smFISH data supports that papillary IC cells frequently express AQP2 and is consistent with the snRNAseq profile of papillary ICs for the expression of AQP2 and SLC26A7 in panel (a). The boxed cells in the corresponding merge panels are enlarged in the far right (scale bars = 5 μm in the enlarged boxes).

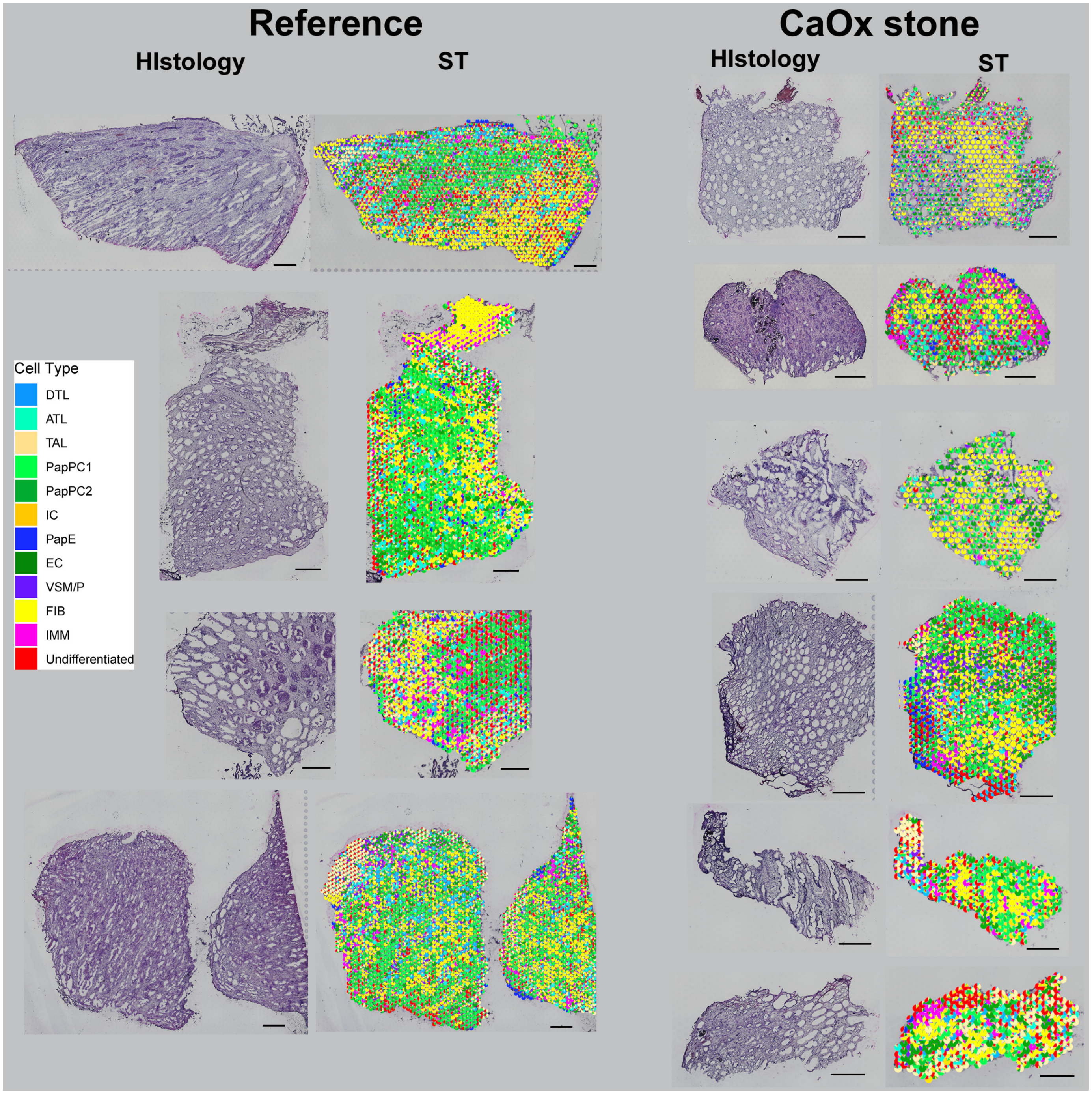

Supplementary Fig. 2: Spatial transcriptomics atlas of the papilla from reference and stone patients- Label transfer and mapping of the snRNAseq cell classes discussed in Figure 1 onto spatial transcriptomic spots in all papilla samples used, with underlying histology shown for each specimen (descending thin limbs- DTL; ascending thin limbs- ATL; thick ascending limbs- TAL; Papillary principal cells- Pap-PC1 and Pap-PC2; intercalated cells-IC; papillary epithelium-PapE; endothelial cells- EC; vascular smooth muscle / pericyte-VSMP; fibroblast-FIB; immune cells-IMM, undifferentiated cells). CaOx= Calcium oxalate. Scale bars = 500 μm.

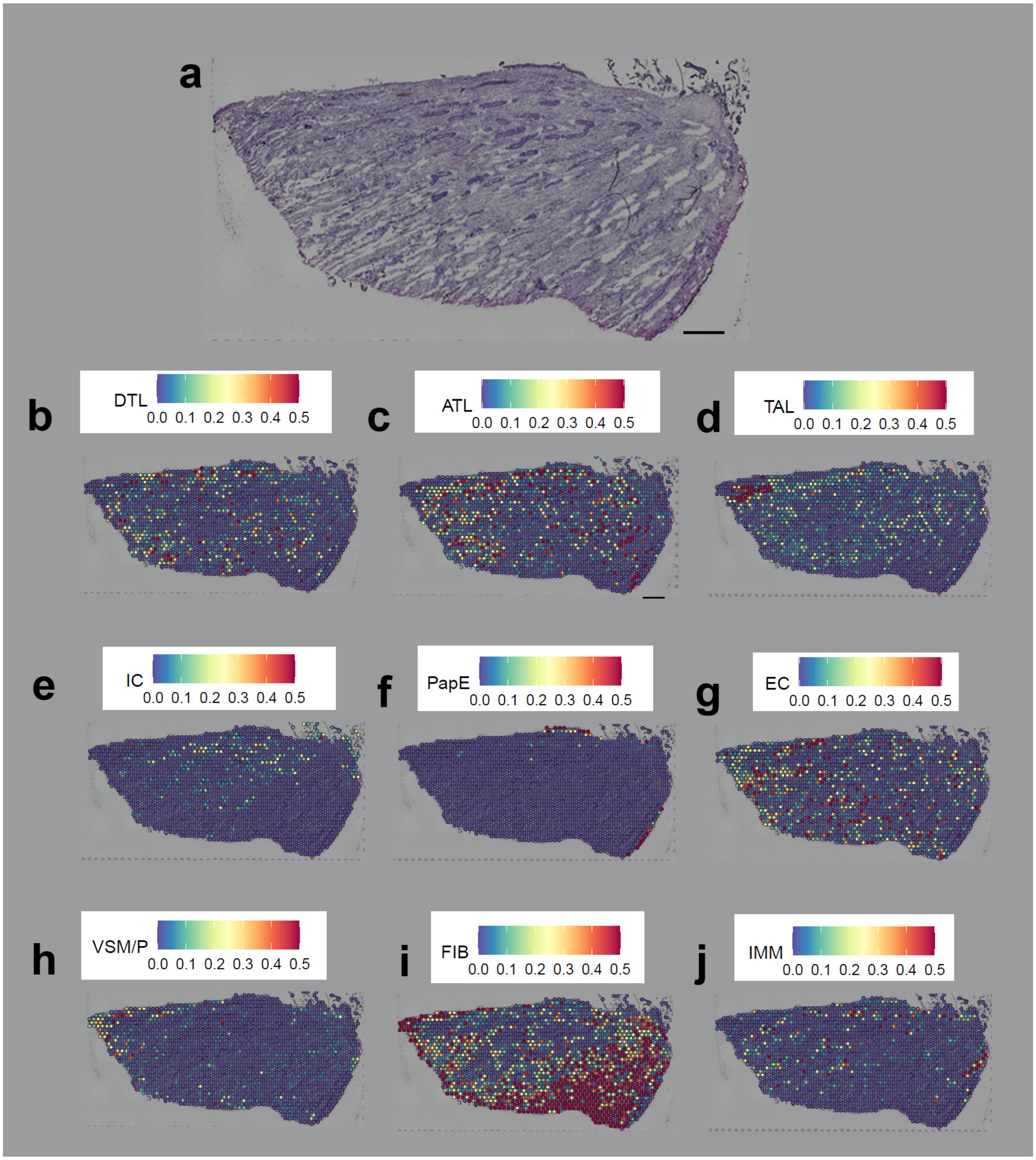

Supplementary Fig. 3: Distribution of various cell signatures for the reference specimen shown in Figure 1. Feature plots showing the mapping of the cell signatures in a representative reference tissue used in Figure 1. (a) shows underlying histology. (b-j) show the distribution of cell signatures indicated in the trop left of each panel. See Supplementary Table 1 for list of abbreviations. Scale bars = 500 μm.

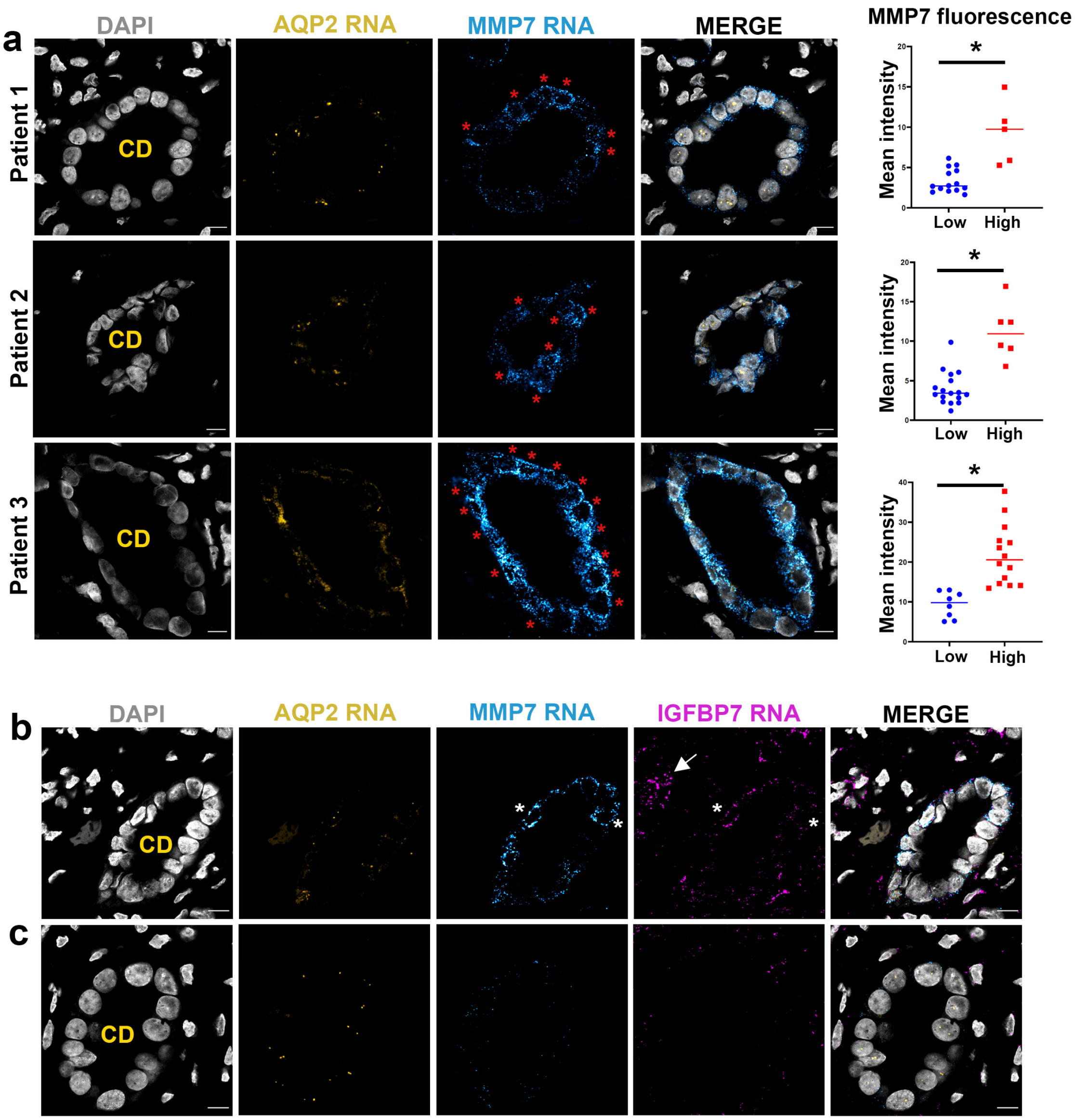

Supplementary Fig. 4: validation of Pap-PC1 and Pap-PC2 cells using single molecular fluorescence in situ hybridization (smFISH) (extended data to Figure 1). (a) smFISH for Aquaporin 2 (*AQP2*) and *MMP7* to distinguish PapPC1 and PapPC2 in papillary specimens from 3 different patients with Calcium oxalate stone disease. Collecting ducts (CD) are morphologically distinct and express *AQP2*. Red asterisks denote cells with high *MMP7* expression which correspond to PapPC2. The *MMP7* fluorescence associated with these cells (n=5 for patient 1; n=6 for patient 2; n= 14 for patient 3) was significantly higher than the rest of the cells (n=14 for patient 1; n=15 for patient 2; n=8 for patient 3) in each corresponding CD (cells manually segmented and fluorescence mean intensity measured using ImageJ. (* statistical significance by two tailed unpaired t-test performed independently for each patient ($p= 0.0001$ patient1, $p< 0.0001$ patient 2, $p= 0.0003$ patient 3)). (b) Few PapPC2 cells (white asterisks) also express *IGFBP7*, which is also consistent with data in Figure 1F. The arrow shows undifferentiated cells outside of CD with high IGFBP7. (c) Collecting duct from the same patient in (b) but different section, not expressing *MMP7* or *IGFBP7*, consisting mostly of PapPC1 cells. Scale bars= 10 μm.

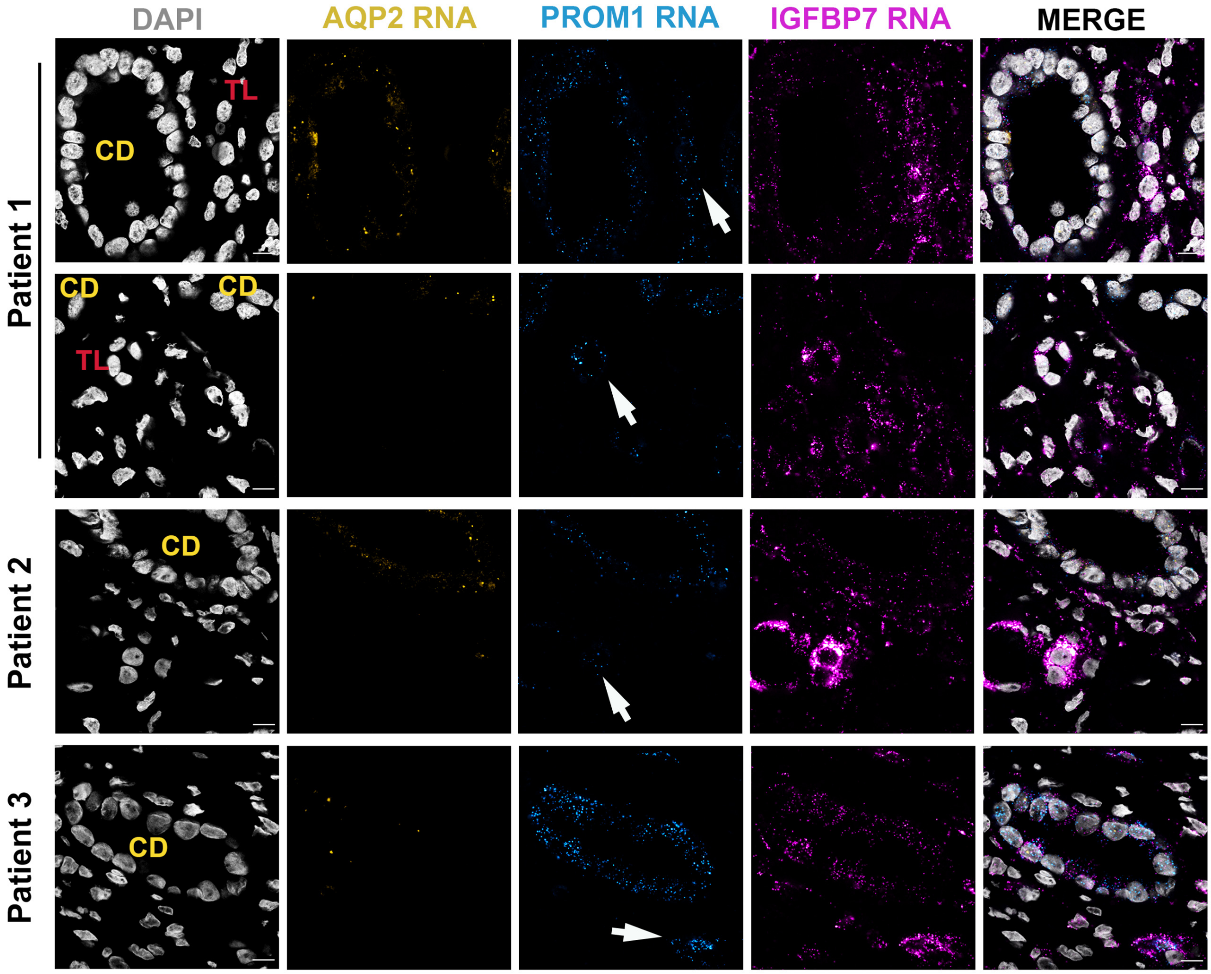

Supplementary Fig. 5: validation of undifferentiated cells using single molecular fluorescence in situ hybridization (smFISH) (extended data to Figure 1). smFISH for *IGFBP7* and *PROM1* was performed on papillary specimens from 3 different patients with Calcium oxalate stone disease. The images highlight undifferentiated cells with high expression of *IGFPB7* and frequently positive for *PROM1* (arrows). These cells localize to the interstitium or in cells with morphology of thin limbs (TL). Scale bars = 10 µm.

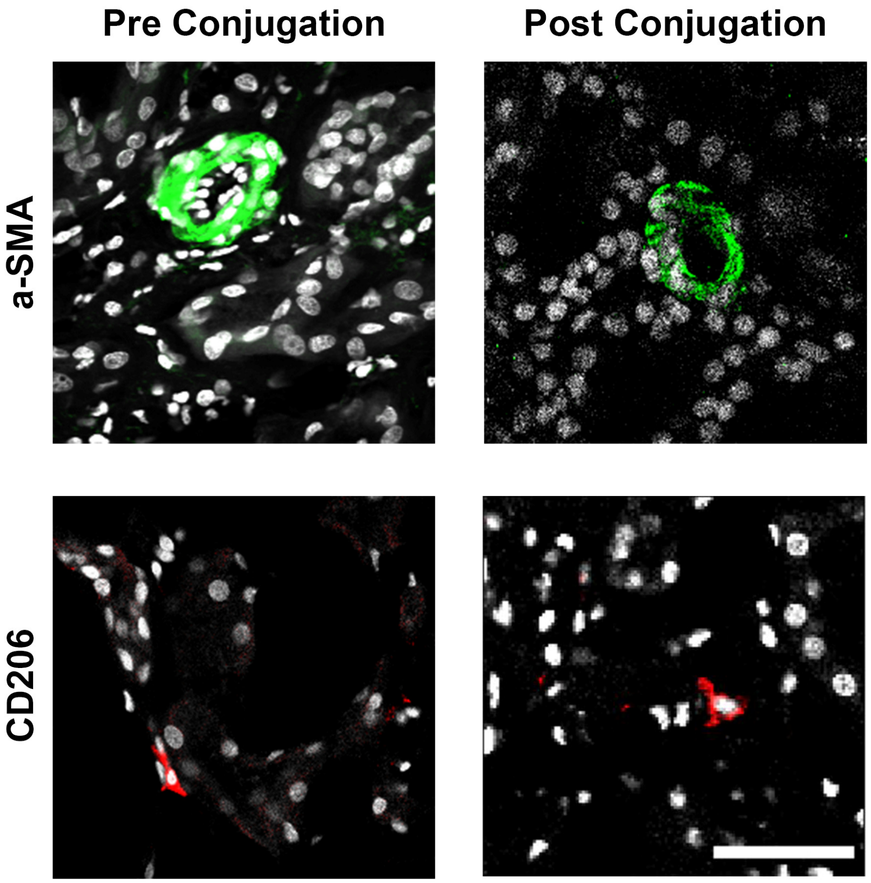

Supplementary Fig. 6: Example of antibody validation before and after conjugation for CODEX imaging. Examples shown here for immunofluorescence staining using antibodies against alpha smooth muscle actin (a-SMA; green), a marker of large vessels and CD206 (in red), a marker of M2 macrophages. Panels on the left represent antibody staining prior to the conjugation of these antibodies to oligonucleotides, while panels on the right represent staining with these antibodies after completed conjugation process. DAPI-stained nuclei are visualized in grey. Scale bar = 50um.

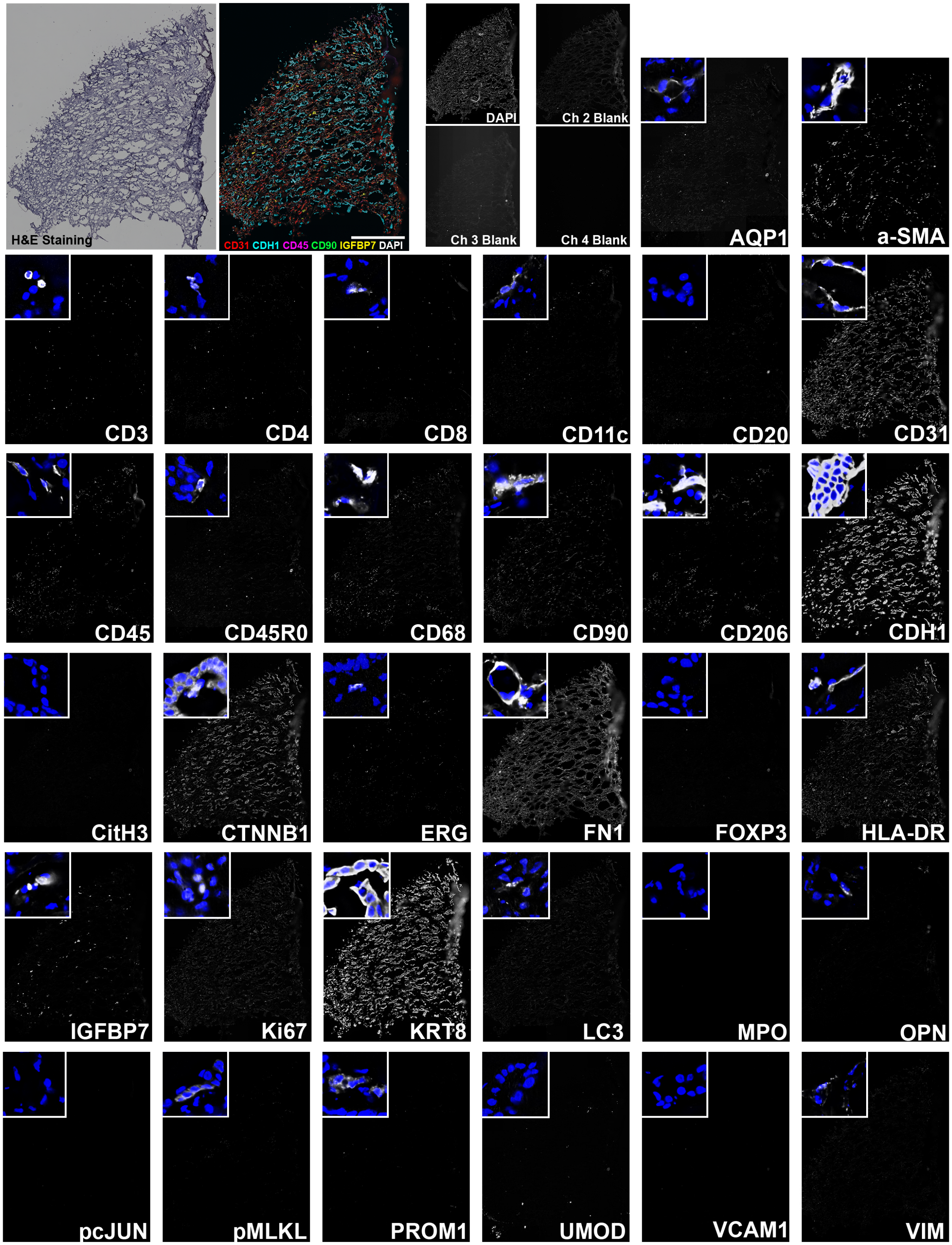
H&E Staining
CD31 CDH1 CD45 CD90 IGFBP7 DAPI
DAPI
Ch 2 Blank
Ch 3 Blank
Ch 4 Blank
AQP1
a-SMA
CD3
CD4
CD8
CD11c
CD20
CD31
CD45
CD45R0
CD68
CD90
CD206
CDH1
CitH3
CTNNB1
ERG
FN1
FOXP3
HLA-DR
IGFBP7
Ki67
KRT8
LC3
MPO
OPN
pcJUN
pMLKL
PROM1
UMOD
VCAM1
VIM

Supplementary Fig. 7: Quality control and cyclical staining of CODEX

Following antibody binding, CODEX imaging goes through cycles of fluorescent probe labeling-imaging and stripping in 4 channels: DAPI- Green (channel2) red (channel 3) and far red (channel 4).

From upper left to Right: Top left panel shows H&E staining of the section that underwent CODEX staining and imaging. A representative image after CODEX is shown to its right (showing merged stains for endothelial marker CD31, distal nephron and CD marker E-cadherin (CDH1), pan leukocyte marker CD45, followed by the mesenchymal/endothelial cell marker CD90 or THY1,injurt marker IGFBP7 and DAPI for nuclei). This is followed by representative images of DAPI alone, and the blanks from channels 2, 3, and 4 that were used for background subtraction during image processing. The panels shown subsequently represent images of each of the antibodies included in the staining panel across the whole reference tissue. Insets show DAPI in blue and antibody staining in white to show staining patterns at the cellular level. Certain markers (CD20, Citrulline H3, FOXP3, MPO, pcJUN, UMOD, VCAM1) were negative in the reference tissue. Cellular level examples are still shown for these antibodies. Scale bar = 1 mm

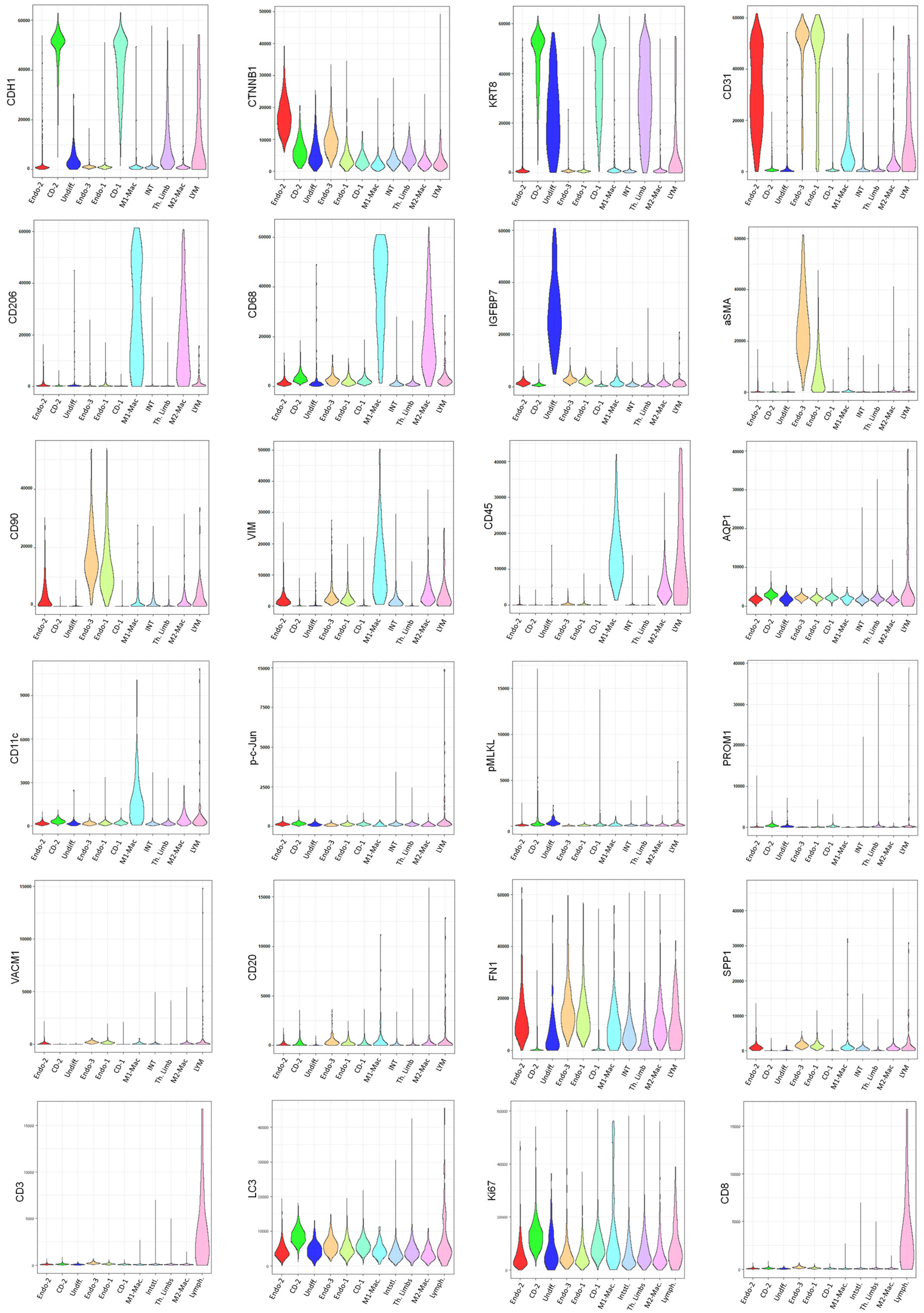
CDH1
CTNNB1
KRT8
CD31
CD206
CD68
IGFBP7
aSMA
CD90
VIM
CD45
AQP1
CD11c
p-c-Jun
pMLKL
PROM1
VACM1
CD20
FN1
SPP1
CD3
LC3
Ki67
CD8
Endo-2
CD-2
Undiff.
Endo-3
Endo-1
CD-1
M1-Mac
INT
Th. Limb
M2-Mac
LYM
Instl.
Th. Limbs
Lymph.

Supplementary Fig. 8: Marker profile for the cell types identified in CODEX analysis in Figure 2

Immunofluorescence intensity profile of the cell classes identified using unsupervised analysis in Figure 2 sorted by cell markers used in CODEX (few markers with low or no signal in the reference tissue were not included). In addition to validating the cell cluster designation based on expression profile, this visualization will also detect any potential cross talk between various markers.

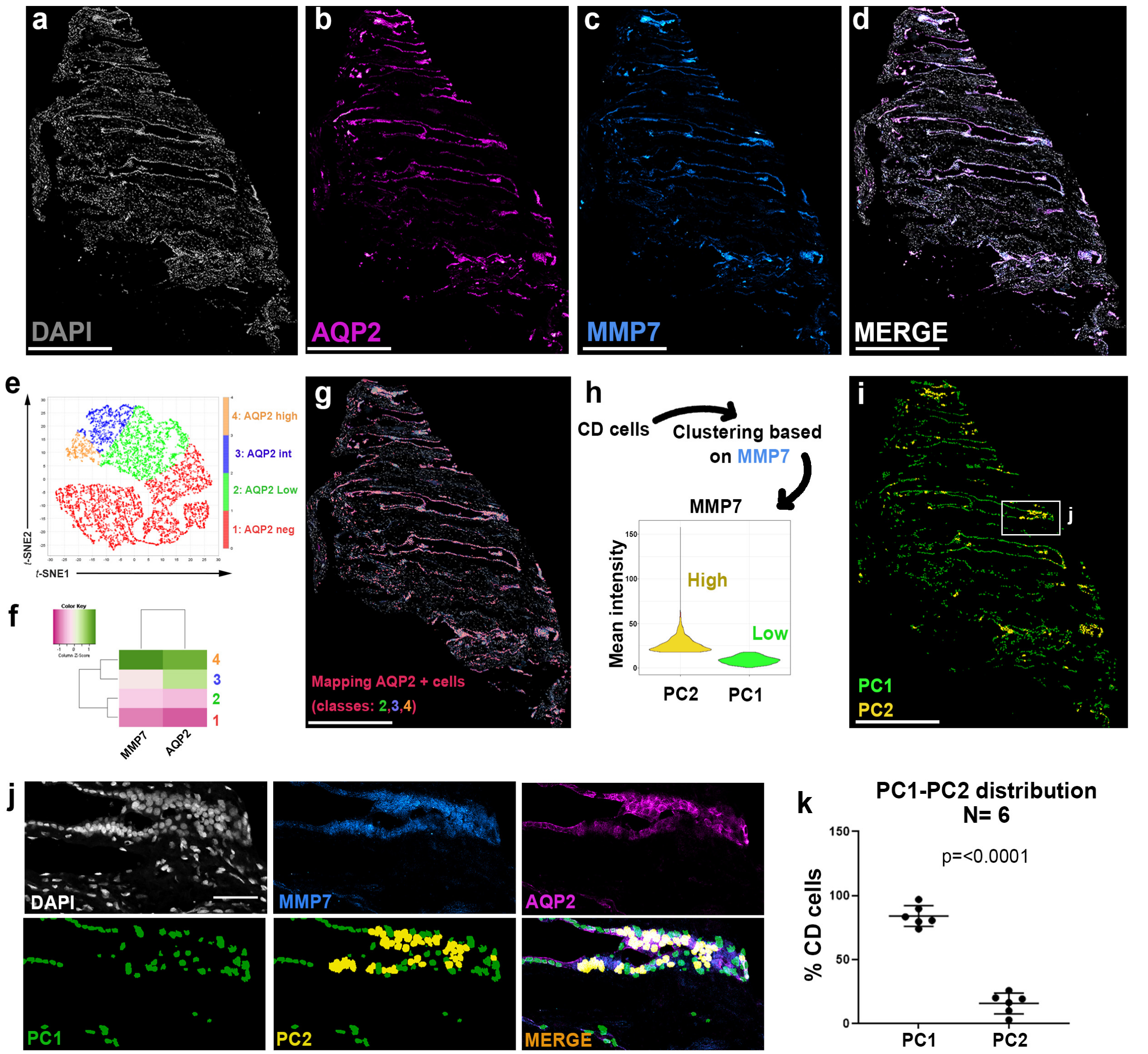

Supplementary Fig. 9: Validation of PapPC1 and PapPC2 cells using large scale 3D imaging and tissue cytometry. (a-d) Representative slices from a confoncal immunofluorescence 3D image volume of a papillary tissue section from a patient with calcium oxalate stone disease stained with AQP2, MPP7 and DAPI. After segmentation with VTEA software, unsupervised analysis was performed based on AQP2 and MMP7 fluorescence (e-f). Collecting duct classes (AQP2+) were distributed in 3 classes based on the fluorescence intensity, and these were mapped for validation in the image volume (g), where nuclear overlays (red) of AQP2+ cells map over AQP2+ collecting ducts. CD cells with high AQP2 expression (class 4) had also high MMP7 expression (f), which is consistent with a PapC2 transcriptomic profile from Figure 1. CD cells were then re-clustered (h) using an unsupervised analysis based on MMP7 fluorescence (MMP7 high and MMP7 low classes), and these were mapped back using nuclear overlays to CDs in the image volume in (i). The boxed area in (i) is enlarged in (j),

showing the staining of AQP2 and MMP7 and the distribution of PapPC1 and PapPC2 (using nuclear overlays) within on CD. (k) Similar analysis was done on 5 other samples (n=3 reference and n=3 stone independent specimens in total), showing consistent distribution and a higher proportion of PapPC1 across samples ($p < 0.0001$ for a difference in proportions of PapPC1 vs PapPC2 using a two tailed paired test). Scale bars in a-d, g, i = 500 μm. Scale bar in j = 50 um. Mean (large bar) and standard deviation (flanking small bars) are shown for each group in (k).

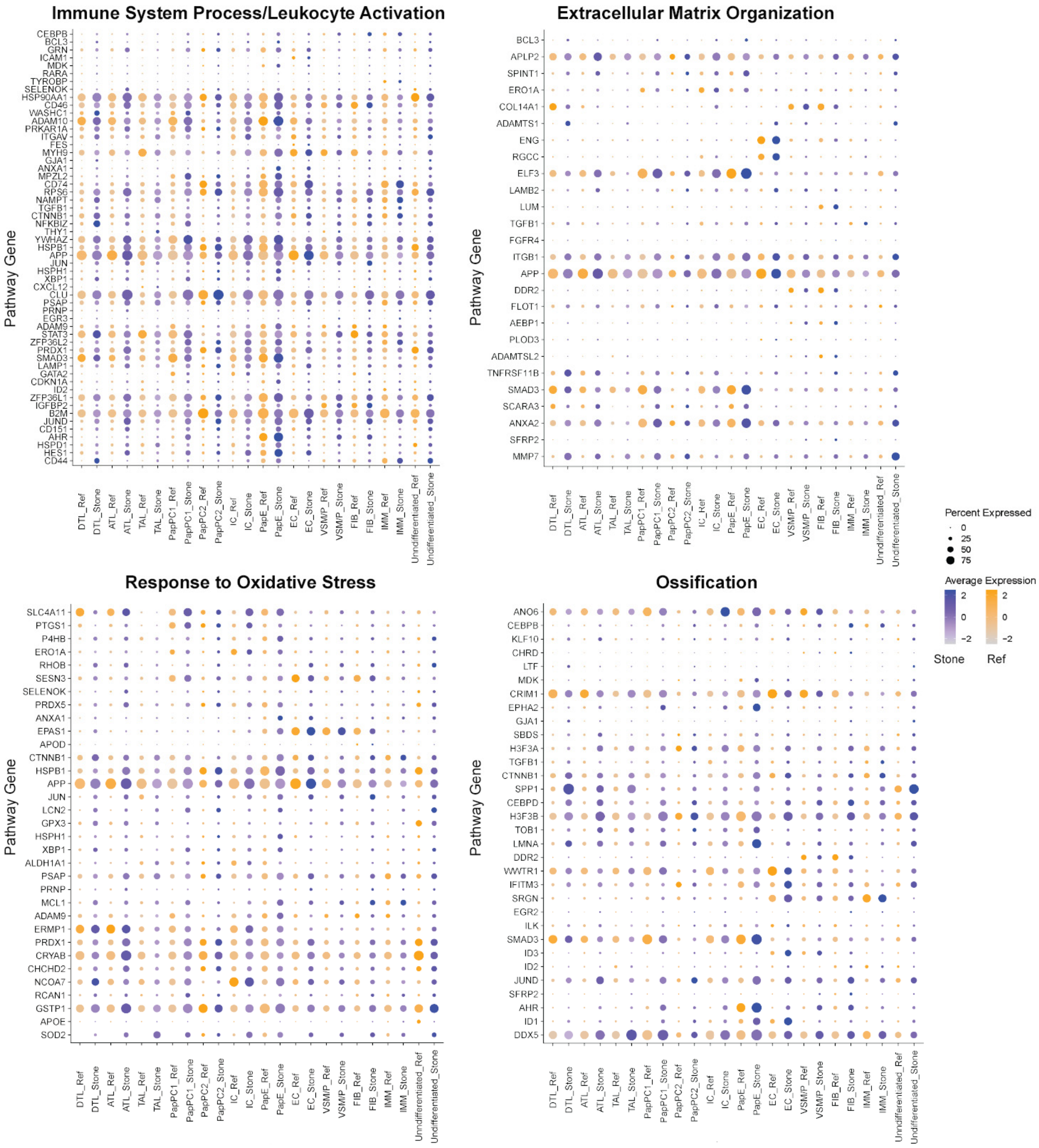

Supplementary Fig. 10: Cell type expression of select genes from pathways enriched in stone versus reference patient papilla biopsies. Genes in relevant pathways (Extracellular Matrix Organization-GO:0030198, Leukoctye Activation-GO:0045321, Ossification-GO:0001503 and Response to Oxidative Stress: GO:0006979) that were significantly increased in all stone biopsies as detected by snRNAseq analysis were compared by dot plot across cell types identified based on their transcriptomic signatures relative to the snRNAseq atlas. Undiff. = undifferentiated; DTL = descending thin limb; ATL = ascending thin limb; TAL = thick ascending limb; PC = principal cell; IC = intercalated cell; PapE = papillary epithelium; EC = epithelial cell; VSM/P = vascular smooth muscle cell; FIB = fibroblast; IMM = immune cell.

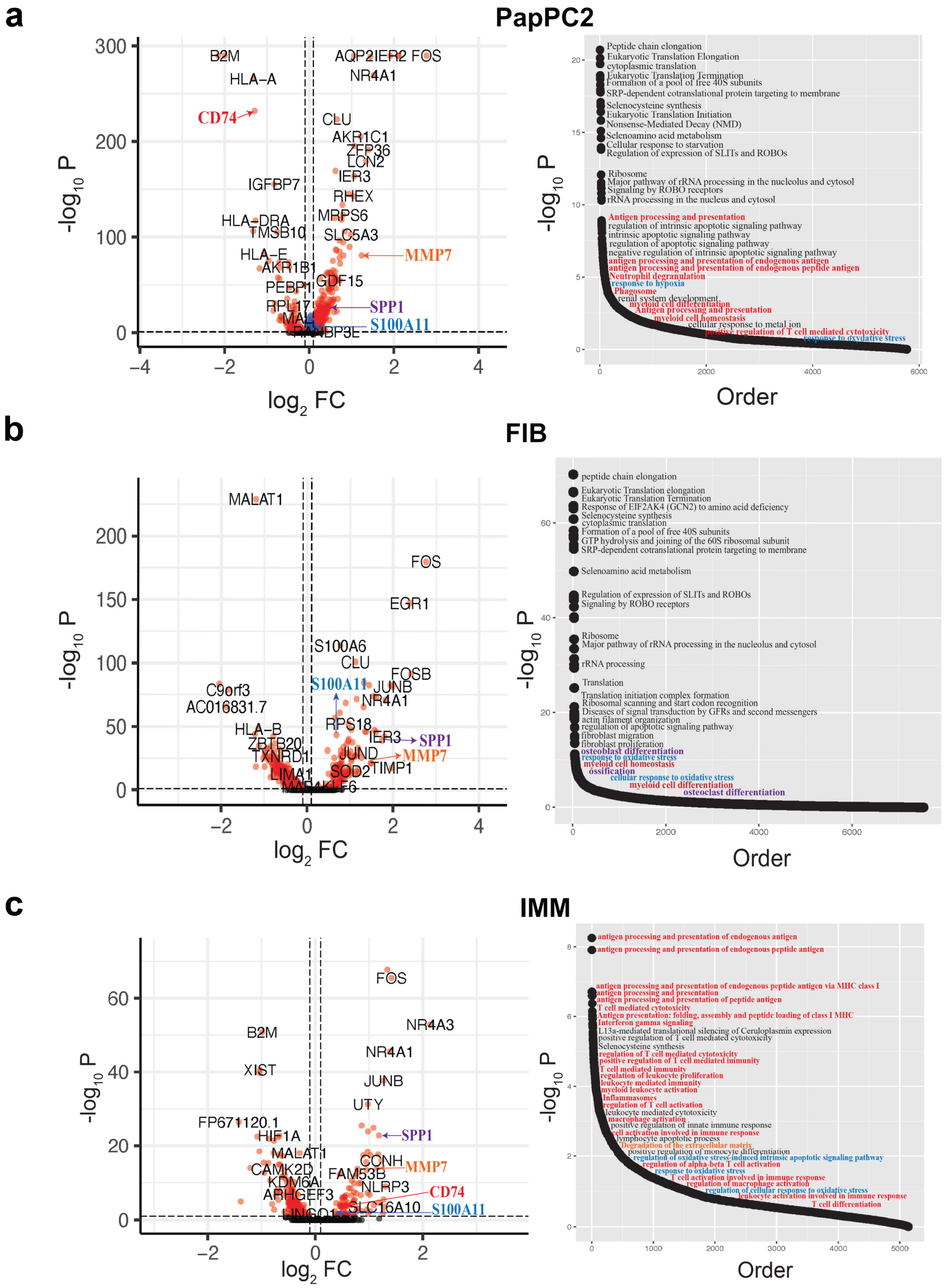

Supplementary Fig. 11: Differentially expressed genes (DEGs) and pathways for various cell types in stone vs. reference samples (extended data for Figure 3). (a), (b) and (c) correspond to PapPC2, fibroblasts and immune cells, respectively. Two tailed Wilcoxon rank sum testing was used to define DEGs. Differentially enriched pathways were determined using overrepresentation testing, as described in methods.[3, 4]

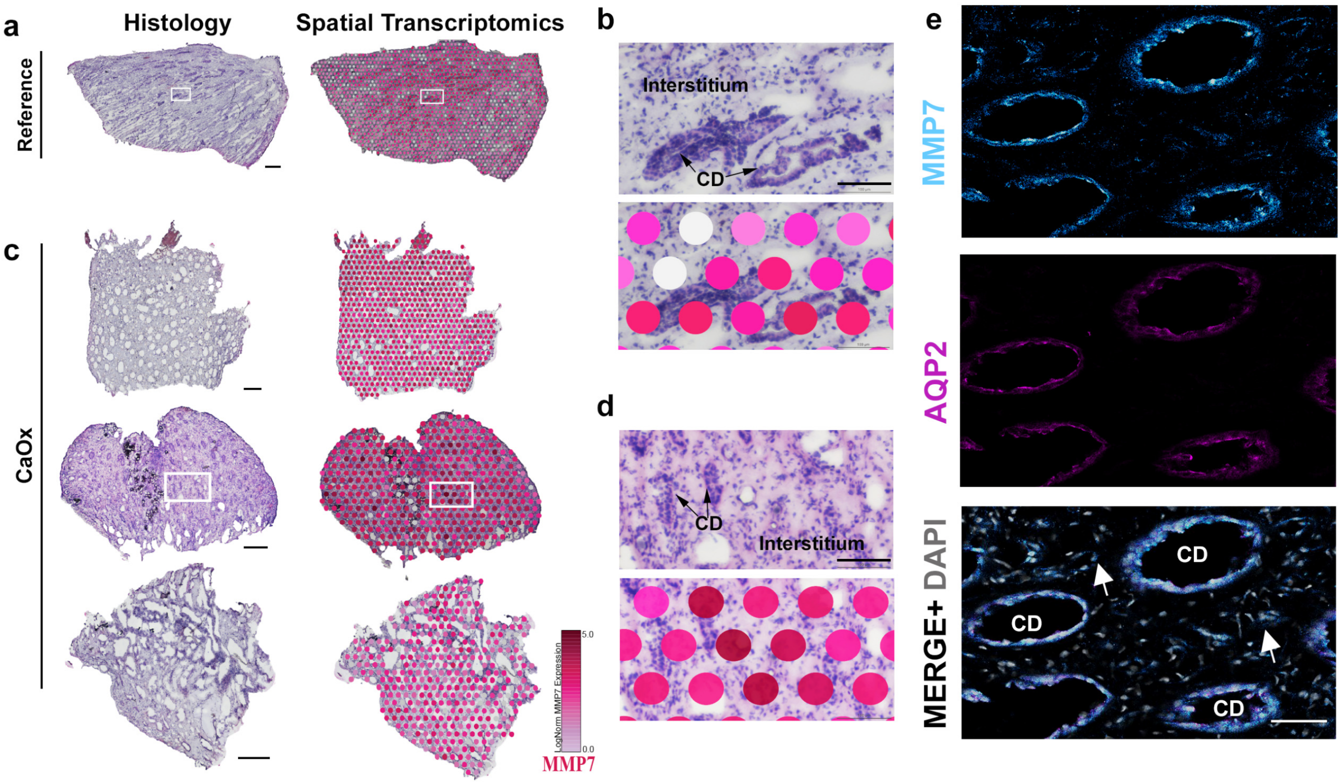

Supplemental Figure 12: MMP7 expression in the papilla. Spatial transcriptomics analysis comparing control (Reference) and three different CaOx stone patient biopsies. In reference tissue (a), MMP7 expression is localized predominantly to collecting ducts (CD) (b). In stone disease, MMP7 expression is diffusely increased and encompasses various papillary cells and structures, which is consistent with the snRNAseq expression and the expression signature mapping on ST (Figure 3). Scales bars in (a) and (c): 0.5 mm for reference and top stone sample, 0.25 mm for other 2 specimen; for (b) and (d): 0.1 mm. (e) Immunofluorescence confocal imaging for AQP2 and MMP7 in a papilla sample from a stone patient confirms the predominant localization of MMP7 to CDs. A less intense signal could also be detected in the surrounding interstitium (arrows). Scale bar in (e) = 10 μm

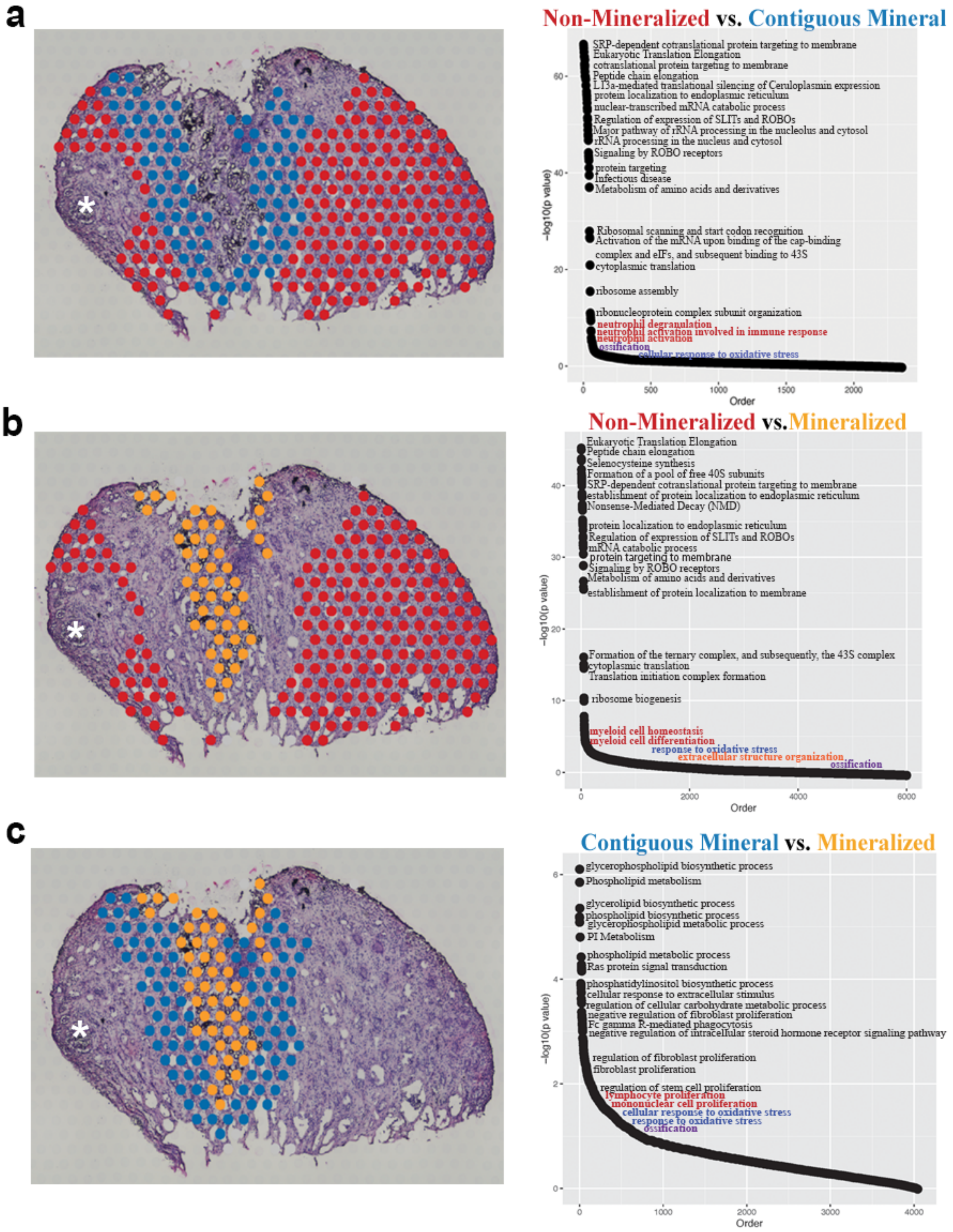

Supplementary Fig. 13: Differentially enriched pathways based on regional analysis and spatial association with mineralization. (a-c) correspond to various comparisons indicated in the top right. Pathways linked to myeloid activation, oxidative stress, matrix remodeling and ossification are colored in red, blue, orange and purple, respectively. Differentially enriched pathways were determined using overrepresentation testing, as described in methods.[3, 4]

Supplementary Table 1- List of abbreviations

TERM	MEANING
CaOX	Calcium oxalate
CD	Collecting duct
IC	Intercalated cell
PC	Principal cell
CODEX	Co-detection by indexing
POD	Podocyte
EC	Endothelial cells
VSM/P	Vascular smooth muscle/pericyte
IMM	Immune
PT	Proximal tubule
PEC	Parietal epithelial cell
DTL	Descending thin limb
ATL	Ascending thin limb
DCT	Distal convoluted tubule
CNT	Connecting tubule
PC	Principal cell
IC	Intercalated cell
PapE	Papillary epithelium
EC	Endothelial cell
FIB	Fibroblast
NEU	Neural cell
PapPC1	Papillary principal cell 1
PapPC2	Papillary principal cell 2
smFISH	Small molecular in situ hybridization
RP	Randall’s plaque
AQP	Aquaporin
MMP	Matrix metalloproteinase

Supplementary Table 2: Papilla biopsy usage in spatial assays and post-sequencing quality control

Papilla ID[1]	Age Range	Sex	Tissue source	Group	snRNA seq	ST	Imaging (3D-IF, CODEX, smFISH)[2]	Number of spots	% Mapping to exons	% Mapping under tissue	Data availability transcriptomics	Data Display
**20-0031**	60-65	M	DecDon	Ref			3D-IF					Suppl. Fig 9
**20-0032**	46-50	F	DecDon	Ref			3D-IF					Fig 3k Suppl. Fig 9
**20-0034**	36-40	F	DecDon	Ref		X	3D-IF	2438	0.92	0.83	GSE 231630	Figs 1, 3I, 6, Suppl. Figs 2, 3
**20-0045**	46-50	M	DecDon	Ref			3D-IF					Fig 3K Suppl. Fig 9
**20-0046**	46-50	M	DecDon	Ref		X	3D-IF Codex	2468	0.50	0.93	GSE 231630	Figs 2, 3I Suppl. Fig 2
**20-0051**	56-60	F	DecDon	Ref		X	3D-IF	1386	0.56	0.36	GSE 231630	Fig 6, Suppl. Fig 2
**20-0052**	61-65	F	DecDon	Ref		X	3D-IF	1376	0.56	0.45	GSE 231630	Fig 6, Suppl. Figs 2, 9
**21-0053**	30-35	F	DecDon	Ref			3D-IF					Fig 6
**K1900387**	66-70	M	DecDon	Ref	X						GSE 183279 HuBMAP[3]	Fig 1, Fig 3
**K2000094**	56-60	F	DecDon	Ref	X						GSE 183279 HuBMAP[4]	Fig 1, Fig 3, Fig 6
**K2100041**	61-65	M	Nx	Ref	X						HuBMAP[5]	Fig 1, Fig 3
**K2100055**	61-65	F	Nx	Ref	X						HuBMAP[6]	Fig 1, Fig 3
**K2100223**	56-60	F	Nx	Ref	X						HuBMAP[7]	Fig 1, Fig 3
**KRP428**	51-55	F	Biopsy	CaOx		X	3D-IF Codex	817	0.53	0.53	GSE 231630	Fig 3E-H, Fig 3I, Fig4, Fig 5, Suppl. Fig 2
**KRP429**	36-40	M	Biopsy	CaOx	X	X	3D-IF	1085	0.82	0.79	GSE 231630	Figs 1, 3A-B, 3I, 6, Suppl. Fig 2
**KRP436**	41-45	M	Biopsy	CaOx			3D-IF					Fig 6
**KRP449**	61-65	F	Biopsy	CaOx		X	smFISH	939	0.86	0.75	GSE 231630	Fig 3I, Suppl. Figs 1D, 2, 5
**KRP446**	26-30	M	Biopsy	CaOx	X	X	3D-IF smFISH	656	0.49	0.49	GSE 183279 HuBMAP[8] GSE 231630	Fig 1P, Fig 3I, Suppl. Figs 1D 4A, 4B-C, 5, 9
**KRP460**	61-65	F	Biopsy	CaOx	X						GSE 183279 HuBMAP[9]	Fig 1, Fig 3
**KRP462**	66-70	F	Biopsy	CaOx	X	X	3D-IF smFISH	262	0.58	0.34	GSE 183279 HuBMAP[10] GSE 231630	Fig 1, Fig 3A-B, Fig 3I, Suppl. Figs 4A, 5, 9
**KRP 463**	31-35	F	Biopsy	CaOx			smFISH					Fig 3I, Suppl. Figs 1D, 4A
**KRP473**	51-55	M	Biopsy	CaOx	X						GSE 231630	Fig 1, Fig 3
**KRP475**	61-65	F	Biopsy	CaOx		X	3D-IF	229	0.58	0.12	GSE 231630	Fig 3I, Suppl. Figs 2, 9
**KRP478**			Biopsy	CaOx			3D-IF					Fig 6
**K2200020[11]**	46-50	M	DecDon	AKI	X						GSE 231630	Fig 1

[1]A medullary sample (K2100202) was added to the kidney atlas in Figure 1 and uploaded to GSE206306 for data availability. The sample was not included in the table because it is not papilla.

[2]All source data for imaging is available at: doi: 10.5281/zenodo.7653239: https://zenodo.org/record/7653239#.ZGIuGc7MKUk

[3-10] Data available through HuBMAP at: [3]https://portal.hubmapconsortium.org/browse/donor/ab8258a97e0820c294d1f0ba2d261f61, [4]https://portal.hubmapconsortium.org/browse/donor/b8f375d33daa5228782abd838d851b8d, [5]https://portal.hubmapconsortium.org/browse/donor/d876de578e9d8c2ce2dcd7c1bbb00681, [6]https://portal.hubmapconsortium.org/browse/donor/9d3c93a2e2d0e81b0f18dd515739a42f, [7]https://portal.hubmapconsortium.org/browse/donor/dfd0352b60504b321899f738c860e3fc, [8]https://portal.hubmapconsortium.org/browse/donor/613776ac210f65a63462ce6f926f4557, [9]https://portal.hubmapconsortium.org/browse/donor/5c4208e2f2715c56ee8dc82b5f1b90c0, 10https://portal.hubmapconsortium.org/browse/donor/e742ac485a0614e4114a26886ed0b444.

[11]Acute Kidney Injury papillary sample included in the cell type mapping but excluded in downstream comparisons between reference and stone papilla.

ST = spatial transcriptomics; 3D-IF = 3-dimensional immunofluorescence imaging, CODEX= codetection by Indexing, smFISH= single molecule fluorescence in situ hybridization, DecDon= Deceased donor, Nx= Tumor nephrectomy, CaOX= Calcium Oxalate stone former, Ref= Reference non-stone former, AKI= Acute Kidney Injury with Blood Urea Nitrogen above 100 mg/dl.

Supplementary Table 3: antibodies used in CODEX assay

Antibody	Significance	Clone	Supplier	Catalog Number
Ki67	Proliferating cells	B56	Akoya	4250019
CD3	Pan T cells	UCHT1	Akoya	4350008
CD4	CD4+ T cells	SK3	Akoya	4350010
CD8	CD8+ t cells	SK1	Akoya	4150004
CD11c	resident dendritic cells	S-HCL-3	Akoya	4350012
CD31	endothelial cells	WM59	Akoya	4250009
CD20	B cells	L26	Akoya	4150018
CD45	pan leukocyte markers	HI30	Akoya	4150003
CD45RO	memory T cells	UCHL1	Akoya	4250023
HLA-DR	antigen presenter cells	L243	Akoya	4250006
CD90 (THY1)	PT, fibroblasts, activated endothelial cells	SE10	Akoya	4150021
E-cadherin (CDH1)	Distal nephron, CD	4A2C7	Akoya	4250021
b-catenin (CTNNB1)	Pan tubular epithelium	12F7	Akoya	4450036
Cytokeratin8 (KRT8)	Loop of Henle, distal nephron and CD	TS1	NovusBio	NBP2-34501-0.1mg
Uromodulin	TAL	Polyclonal	R&D	AF5144
a-sma	myofibroblast, arterioles	1A4	Invitrogen	14-9760-82
PROM1 (CD133)	fibrosis	AC133	Miltenyi Biotec	130-090-422
MPO	neutrophils	Polyclonal	Abcam	ab9535
CD68	activated macrophages	KP1	ThermoFisher	14-0688-82
IGFBP7	injury	Polyclonal	Acris/Origene	AP01109PU-S
p- c-Jun	stress kinase pathway	D47G9	Cell Signalling	3270BF
CD206	M2	Polyclonal	R&D	AF2534

SPP1	Osteopontin /OPN	AKm2A1	Santa Cruz	sc21742
ERG	Endothelial Nuclei	EPR3864	Abcam	ab92513
AQP1	PT, TDL	1/22	Santa Cruz	sc-32737-X
Citruline H3	netosis	7C10	Acris/Origene	AM10179PU-N
Vimentin	Fibroblasts	RV202	BD Pharmingen	550513
FOXP3	injury	236A/E7	Thermo Fisher	14-4777-82
VCAM1	non-repairing epithelial cells	EPR5047	Abcam	ab271899
Phosphor-MLKL	necroptosis	D6H3V (S358)	Cell Signaling	91689BF
Fibronectin	Injury, pre-collagen	F1	Abcam	ab271831
LC3	autophagy	Polyclonal	Sigma Aldrich	L8918-25UL

Antibodies purchased from Akoya were conjugated by vendor. Antibodies from other vendors were conjugated in-house using Akoya conjugation kits as described in methods

Supplementary Table 4: Clinical summary of urine donors for MMP7/9 studies.

	**Normal** N= 20	**Non-Active SF** N= 18	**Active SF** N= 18	***P***
**Age**	41 +/- 8.9	39 +/- 9.0	47 +/- 11.9	ns
**Sex** (% male)	50	67	40	ns
**Race** (% white, non-hispanic)	100	100	100	ns
**EGFR** (ml/min)	93.2 +/- 19.7	98.10 +/- 18.9	88.9 +/- 22.8	ns
**Serum Creatinine** (mg/dl)	0.92 +/- 0.09	0.93 +/- 0.12	1.00 +/- 0.45	ns
**Urine Creatinine** (mg/dl)	152.3 +/- 64.0	119.9 +/- 57.0	121.3 +/- 74.7	ns
**Diabetes** (%)	0	0	5.6	N/A
**HTN** (%)	0	0	0	N/A
**Cardiac Disease** (%)	0	0	0	N/A

Except for proportions, values represent mean ± standard deviation. Comparisons were done using a two-tailed ANOVA.

EGFR= Estimated glomerular filtration rate

SUPPLEMENTARY REFERENCES

1. Petrovic S, *et al.* SLC26A7: a basolateral Cl-/HCO3- exchanger specific to intercalated cells of the outer medullary collecting duct. *Am J Physiol Renal Physiol* **286**, F161-169 (2004).

2. Lake BB, *et al.* An atlas of healthy and injured cell states and niches in the human kidney. *bioRxiv*, 2021.2007.2028.454201 (2021).

3. Yu G, He QY. ReactomePA: an R/Bioconductor package for reactome pathway analysis and visualization. *Mol Biosyst* **12**, 477-479 (2016).

4. Yu G, Wang LG, Han Y, He QY. clusterProfiler: an R package for comparing biological themes among gene clusters. *OMICS* **16**, 284-287 (2012).

KPMP consortium members

Name	E-mail	Site
Stewart H. Lecker	slecker@bidmc.harvard.edu	Beth Israel Deaconess
Isaac Stillman	istillma@caregroup.harvard.edu	Beth Israel Deaconess
Steve Bogen	sbogen@bostoncellstandards.com	Boston Cell Standards
Afolarin A. Amodu	Afolarin.Amodu@bmc.org	Boston Medical Center
Titlayo Ilori	tilori1@bu.edu	Boston Medical Center
Insa Schmidt	Insa.Schmidt@bmc.org	Boston Medical Center
Shana Maikhor	Shana.Maikhor@bmc.org	Boston Medical Center
Laurence H. Beck	Laurence.Beck@bmc.org	Boston University
Joel M. Henderson	Joel.Henderson@bmc.org	Boston University
Ingrid Onul	Ingrid.Onul@bmc.org	Boston University
Ashish Verma	Ashish.Verma@bmc.org	Boston University
Gearoid M. McMahon	gmmcmahon@bwh.harvard.edu	Brigham & Women's Hospital
M. Todd Valerius	MTVALERIUS@BWH.HARVARD.EDU	Brigham & Women's Hospital
Sushrut Waikar	swaikar@bu.edu	Brigham & Women's Hospital
Astrid Weins	aweins@bwh.harvard.edu	Brigham & Women's Hospital
Mia R. Colona	Mia.Colona@bmc.org	Brigham & Women's Hospital
Anna Greka	agreka@broadinstitute.org	Broad Institute
Nir Hacohen	nhacohen@partners.org	Broad Institute
Paul J. Hoover	phoover@bwh.harvard.edu	Broad Institute
Jamie L. Marshall	jmarshal@broadinstitute.org	Broad Institute
Mark Aulisio	mpa5@case.edu	Case Western Reserve
Yijiang M. Chen	yxc627@case.edu	Case Western Reserve
Andrew Janowczyk	andrew.janowczyk@case.edu	Case Western Reserve
Vidya S. Viswanathan	vxv@case.edu	Case Western Reserve
William S. Bush	wsb36@case.edu	Case Western Reserve University
Dana C. Crawford	dcc64@case.edu	Case Western Reserve University
Anant Madabhushi	axm788@case.edu	Case Western Reserve University
Lakeshia Bush	BUSHL3@ccf.org	Cleveland Clinic
Agustin Gonzalez-Vicente	agustin.gonzalezvicente@case.edu	Cleveland Clinic
Leal Herlitz	herlitl@ccf.org	Cleveland Clinic
Jane Nguyen	nguyenj@ccf.org	Cleveland Clinic
John O'toole	otoolej@ccf.org	Cleveland Clinic
Emilio Poggio	poggioe@ccf.org	Cleveland Clinic
John Sedor	sedorj@ccf.org	Cleveland Clinic
Dianna Sendrey	SENDRED2@ccf.org	Cleveland Clinic
Kassandra Spates-Harden	SPATESK2@ccf.org	Cleveland Clinic

Jonathan Taliercio	TALIERJ@ccf.org	Cleveland Clinic
Leslie Cooperman	COOPERL2@ccf.org	Cleveland Clinic
Stacey Jolly	JOLLYS@ccf.org	Cleveland Clinic
Ellen Palmer	elp76@case.edu	Cleveland Clinic
Petter M. Bjornstad	Petter.Bjornstad@childrenscolorado.org	Colorado
Laura Pyle	laura.pyle@cuanschutz.edu	Colorado
Paul Appelbaum	appelba@nyspi.columbia.edu	Columbia University
Jonathan M. Barasch	jmb4@columbia.edu	Columbia University
Andrew S. Bomback	asb68@columbia.edu	Columbia University
Pietro A. Canetta	pac2004@cumc.columbia.edu	Columbia University
Vivette D. D'Agati	vdd1@columbia.edu	Columbia University
Krzysztof Kiryluk	kk473@cumc.columbia.edu	Columbia University
Ning Shang	ns3026@cumc.columbia.edu	Columbia University
Olivia Balderes	ob2214@cumc.columbia.edu	Columbia University
Karla Mehl	km3246@cumc.columbia.edu	Columbia University
Shweta Bansal	laura.barisoni@duke.edu	Duke University
Theodore Alexandrov	theodore.alexandrov@embl.de	European Molecular Biology Laboratory
Helmut Rennke	hrennke@bwh.harvard.edu	Harvard University
Tarek M. El-Achkar	telachka@iu.edu	Indiana University
Daria Barwinska	dbarwins@iu.edu	Indiana University
Sharon Bledsoe	sbledsoe@iupui.edu	Indiana University
Katy Borner	katy@indiana.edu	Indiana University
Andreas Bueckle	abueckle@indiana.edu	Indiana University
Pierre C. Dagher	pdaghe2@iu.edu	Indiana University
Kenneth W. Dunn	kwdunn@iu.edu	Indiana University
Michael T. Eadon	meadon@iupui.edu	Indiana University
Michael J. Ferkowicz	mferkow@iu.edu	Indiana University
Bruce W. Herr	bherr@indiana.edu	Indiana University
Katherine J. Kelly	kajkelly@iu.edu	Indiana University
Ricardo Melo Ferreira	rimelof@iu.edu	Indiana University
Ellen M. Quardokus	ellenmq@indiana.edu	Indiana University
Elizabeth Record	recorde@indiana.edu	Indiana University
Marcelino Rivera	maeriver@iu.edu	Indiana University
Jing Su	su1@iu.edu	Indiana University
Timothy A. Sutton	tsutton2@iu.edu	Indiana University
James C. Williams, Jr.	jwillia3@iupui.edu	Indiana University
Seth Winfree	winfrees@iu.edu	Indiana University
Steven Menez	smenez1@jhmi.edu	Johns Hopkins University

Chirag R. Parikh	chirag.parikh@jhmi.edu	Johns Hopkins University
Avi Rosenberg	arosen34@jhmi.edu	Johns Hopkins University
Celia P. Corona-Villalobos	pamelacorona@jhmi.edu	Johns Hopkins University
Yumeng Wen	ywen14@jhmi.edu	Johns Hopkins University
Camille Johansen	Camille.Johansen@joslin.harvard.edu	Joslin
Sylvia E. Rosas	sylvia.rosas@joslin.harvard.edu	Joslin Diabetes Center
Neil Roy	neil.roy@joslin.harvard.edu	Joslin Diabetes Center
Jennifer Sun	Jennifer.Sun@joslin.harvard.edu	Joslin Diabetes Center
Mark Williams	mark.williams@joslin.harvard.edu	Joslin Diabetes Center
Richard Knight	rk.reslend@gmail.com	KPMP Patient Partner
Joseph Ardayfio	joseph.ardayfio@gmail.com	KPMP Patient Partner
Keith Brown	kdblawyer@icloud.com	KPMP Patient Partner
Catherine E. Campbell	cec5479@yahoo.com	KPMP Patient Partner
Taneisha Campbell	tcampbell@giftoflifemichigan.org	KPMP Patient Partner
Lynda Hayashi	lyndahayashi@gmail.com	KPMP Patient Partner
Nichole Jefferson	nicholejeff@gmail.com	KPMP Patient Partner
Glenda V. Roberts	glendar@Nephrology.washington.edu	KPMP Patient Partner
John Saul	jbsaul@mac.com	KPMP Patient Partner
Anna Shpigel	blumkinlaw@yahoo.com	KPMP Patient Partner
Christy Stutzke	dzinri@aol.com	KPMP Patient Partner
Jack Bebiak	jackb@gspire.org	KPMP Patient Partner
Robert Koewler	rob.koewler@gmail.com	KPMP Patient Partner
Roy Pinkeney	20shuvee11@gmail.com	KPMP Patient Partner
Evren U. Azeloglu	evren.azeloglu@mssm.edu	Mount Sinai
Jens Hansen	jens.hansen@mssm.edu	Mount Sinai
Cijang He	cijiang.he@mssm.edu	Mount Sinai
Ravi Iyengar	ravi.iyengar@mssm.edu	Mount Sinai
Yuguang Xiong	yuguang.xiong@mssm.edu	Mount Sinai
Pottumarthi Prasad	PPrasad@northshore.org	Northshore
Anand Srivastava	anand.srivastava@northwestern.edu	Northwestern
Sethu M. Madhavan	Sethu.Madhavan@osumc.edu	Ohio State University
Samir Parikh	samir.parikh@osumc.edu	Ohio State University
Brad Rovin	brad.rovin@osumc.edu	Ohio State University
John P. Shapiro	John.Shapiro@osumc.edu	Ohio State University
Christopher R. Anderton	christopher.anderton@pnnl.gov	Pacific Northwest National Laboratories
Ljiljana Pasa-Tolic	ljiljana.pasatolic@pnnl.gov	Pacific Northwest National Laboratories
Dusan Velickovic	dusan.velickovic@pnnl.gov	Pacific Northwest National Laboratories

Jessica Lukowski	jessica.lukowski@pnnl.gov	Pacific Northwest National Laboratories
George (Holt) Oliver	george.oliver@phhs.org	Parkland
Rachel Sealfon	rsealfon@flatironinstitute.org	Princeton University
Olga Troyanskaya	ogt@genomics.princeton.edu	Princeton University
Aaron Wong	awong@flatironinstitute.org	Princeton University
Katherine R. Tuttle	katherine.tuttle@providence.org	Providence Health
Ari Pollack	ari.pollack@seattlechildrens.org	Seattle Children's Hospital
Yury Goltsev	goltsev@stanford.edu	Stanford University
Nicholas Lucarelli	njlucare@buffalo.edu	SUNY Buffalo
Pinaki Sarder	pinaki.sarder@ufl.edu	SUNY Buffalo
Blue B. Lake	blake@altoslabs.com	UC San Diego
Kun Zhang	kzhang@eng.ucsd.edu	UC San Diego
Patrick Boada	Patrick.Boada@ucsf.edu	UC San Francisco
Zoltan G. Laszik	Zoltan.Laszik@ucsf.edu	UC San Francisco
Minnie Sarwal	Minnie.Sarwal@ucsf.edu	UC San Francisco
Tara Sigdel	tara.sigdel@ucsf.edu	UC San Francisco
Kavya Anjani	Kavya.Anjani@ucsf.edu	UC San Francisco
Tariq Mukatash	Tariq.Mukatash@ucsf.edu	UC San Francisco
Rita R. Alloway	ALLOWARR@UCMAIL.UC.EDU	University of Cincinnati
Ashley R. Burg	Ashley.Burg@cchmc.org	University of Cincinnati
Paul J. Lee	leep7@ucmail.uc.edu	University of Cincinnati
Adele Rike	rikea@ucmail.uc.edu	University of Cincinnati
Tiffany Shi	tiffany.shi@cchmc.org	University of Cincinnati
E. Steve Woodle	WOODLEES@UCMAIL.UC.EDU	University of Cincinnati
Heather Ascani	ascanihk@med.umich.edu	University of Michigan
Ulysses GJ. Balis	ulysses@med.umich.edu	University of Michigan
Victoria M. Blanc	vmb@med.umich.edu	University of Michigan
Ninive C. Conser	costan@med.umich.edu	University of Michigan
Sean Eddy	seaneddy@med.umich.edu	University of Michigan
Yougqun He	yongqunh@med.umich.edu	University of Michigan
Jeffrey B. Hodgin	jhodgin@umich.edu	University of Michigan
Matthias Kretzler	kretzler@med.umich.edu	University of Michigan
Chrysta Lienczewski	boridley@med.umich.edu	University of Michigan
Jinghui Luo	jinghui@umich.edu	University of Michigan
Laura H. Mariani	lmariani@med.umich.edu	University of Michigan
Rajasree Menon	rajmenon@med.umich.edu	University of Michigan
Edgar Otto	eotto@med.umich.edu	University of Michigan
Jennifer Schaub	schaubj@med.umich.edu	University of Michigan

Becky Steck	roesch@med.umich.edu	University of Michigan
Renee Frey	frenee@med.umich.edu	University of Michigan
Michele M. Elder	mie1@pitt.edu	University of Pittsburgh
Daniel E. Hall	hallde@upmc.edu	University of Pittsburgh
Raghavan Murugan	muruganr@ccm.upmc.edu	University of Pittsburgh
Paul M. Palevsky	palevsky@pitt.edu	University of Pittsburgh
Parmjeet Randhawa	randhawapa@upmc.edu	University of Pittsburgh
Matthew Rosengart	rosengartmr@upmc.edu	University of Pittsburgh
Mitchell Tublin	tublme@upmc.edu	University of Pittsburgh
Tina Vita	vitatm@upmc.edu	University of Pittsburgh
Matthew Gilliam	MJG136@pitt.edu	University of Pittsburgh
John A. Kellum	kellum@pitt.edu	University of Pittsburgh
James Winters	wintersj4@upmc.edu	University of Pittsburgh
Charles E. Alpers	calp@u.washington.edu	University of Washington
Ashley Berglund	bergla@uw.edu	University of Washington
Brooke Berry	bberry3@uw.edu	University of Washington
Kristina N. Blank	blankk@u.washington.edu	University of Washington
Jonas Carson	jcarson@u.washington.edu	University of Washington
Stephen Daniel	sdaniel@uw.edu	University of Washington
Ian H. De Boer	IDeBoer@Nephrology.washington.edu	University of Washington
Ashveena L. Dighe	ashveena@Nephrology.washington.edu	University of Washington
Frederick Dowd	dowdf@u.washington.edu	University of Washington
Jonathan Himmelfarb	jhimmelfarb@nephrology.washington.edu	University of Washington
Andrew N. Hoofnagle	ahoof@uw.edu	University of Washington
Christine Limonte	climonte@uw.edu	University of Washington
Robyn L. McClelland	rmcclell@u.washington.edu	University of Washington
Natalya Sarkisova	nasark@kri.washington.edu	University of Washington
Sean D. Mooney	sdmooney@u.washington.edu	University of Washington
Kasra Rezaei	krezaei@uw.edu	University of Washington
Stuart Shankland	Sshankland@Nephrology.washington.edu	University of Washington
Jamie Snyder	jas1208@uw.edu	University of Washington
Ruikang Wang	wangr@uw.edu	University of Washington
Stephanie M. Grewenow	stephaniegrewenow@gmail.com	University of Washington
Christopher Park	cpark3@nephrology.washington.edu	University of Washington
Adam Wilcox	abwilcox@uw.edu	University of Washington
Kayleen Williams	kmfw@u.washington.edu	University of Washington
Shweta Bansal	bansals3@uthscsa.edu	UT Health San Antonio

Richard Montellano	montellano@uthscsa.edu	UT Health San Antonio
Annapurna Pamreddy	pamreddy@uthscsa.edu	UT Health San Antonio
Kumar Sharma	sharmak3@uthscsa.edu	UT Health San Antonio
Manjeri Venkatachalam	venkatachal@uthscsa.edu	UT Health San Antonio
Hongping Ye	yeh1@uthscsa.edu	UT Health San Antonio
Guanshi Zhang	zhangg3@uthscsa.edu	UT Health San Antonio
S. Susan Hedayati	susan.hedayati@utsouthwestern.edu	UT Southwestern
Asra Kermani	Asra.Kermani@UTSouthwestern.edu	UT Southwestern
Christopher Y. Lu	Christopher.lu@utsouthwestern.edu	UT Southwestern
R. Tyler Miller	tyler.miller@utsouthwestern.edu	UT Southwestern
Orson W. Moe	orson.moe@utsouthwestern.edu	UT Southwestern
Jiten Patel	Jiten.Patel@UTSouthwestern.edu	UT Southwestern
Anil Pillai	Anil.pillai@utsouthwestern.edu	UT Southwestern
Kamalanathan Sambandam	KSAMBANDAM@UTSouthwestern.edu	UT Southwestern
Jose Torrealba	jose.torrealba@utsouthwestern.edu	UT Southwestern
Robert D. Toto	robert.toto@utsouthwestern.edu	UT Southwestern
Miguel Vazquez	miguel.vazquez@utsouthwestern.edu	UT Southwestern
Nancy Wang	Zhengnan.Wang@UTSouthwestern.edu	UT Southwestern
Dianbo Zhang	Dianbo.Zhang@UTSouthwestern.edu	UT Southwestern
Simon C. Lee	simoncraddock.lee@utsouthwestern.edu	UT Southwestern
Harold Park	Harold.park@utsouthwestern.edu	UT Southwestern
Natasha Wen	yu.wen@utsouthwestern.edu	UT Southwestern
Richard M. Caprioli	richard.m.caprioli@Vanderbilt.Edu	Vanderbilt
Nathan Patterson	nathan.h.patterson@vanderbilt.edu	Vanderbilt
Kavya Sharman	kavya.sharman@Vanderbilt.Edu	Vanderbilt
Jeffrey M. Spraggins	jeff.spraggins@Vanderbilt.Edu	Vanderbilt
Raf Van de Plas	raf.vandeplas@tudelft.nl	Vanderbilt
Jeanine Basta	jbasta@wustl.edu	Washington University St. Louis
Sabine M. Diettman	sdietmann@wustl.edu	Washington University St. Louis
Joseph P. Gaut	jpgaut@wustl.edu	Washington University St. Louis
Sanjay Jain	jains@dom.wustl.edu	Washington University St. Louis
Michael I. Rauchman	mrauchma@wustl.edu	Washington University St. Louis
Anitha Vijayan	avijayan@wustl.edu	Washington University St. Louis
Lloyd G. Cantley	lloyd.cantley@yale.edu	Yale University
Vijaykumar R. Kakade	vijayakumar.kakade@yale.edu	Yale University
Dennis Moledina	dennis.moledina@yale.edu	Yale University

Melissa M. Shaw	melissa.m.shaw@yale.edu	Yale University
Ugochukwu Ugwuowo	ugochukwu.ugwuowo@yale.edu	Yale University
Francis P. Wilson	francis.p.wilson@yale.edu	Yale University
Tanima Arora	Tanima.arora@yale.edu	Yale University
