# Supplementary material for: A spatially anchored transcriptomic atlas of the human kidney papilla identifies significant immune injury in patients with stone disease
Source: Nat Commun. 2023 Jul 19;14:4140. doi: 10.1038/s41467-023-38975-8 (PMC10356953; doi:10.1038/s41467-023-38975-8)
Supplement: Supplementary file 3 — Reporting Summary [file 41467_2023_38975_MOESM3_ESM.pdf]

nature portfolio

Corresponding author(s): Sanjay Jain, Michael Eadon, James Williams and Tarek M El-Achkar

Last updated by author(s): May 15, 2023

Reporting Summary

Nature Portfolio wishes to improve the reproducibility of the work that we publish. This form provides structure for consistency and transparency in reporting. For further information on Nature Portfolio policies, see our Editorial Policies and the Editorial Policy Checklist.

Statistics

For all statistical analyses, confirm that the following items are present in the figure legend, table legend, main text, or Methods section.

n/a	Confirmed	
☐	☒	The exact sample size (*n*) for each experimental group/condition, given as a discrete number and unit of measurement
☐	☒	A statement on whether measurements were taken from distinct samples or whether the same sample was measured repeatedly
☐	☒	The statistical test(s) used AND whether they are one- or two-sided<br>*Only common tests should be described solely by name; describe more complex techniques in the Methods section.*
☒	☐	A description of all covariates tested
☐	☒	A description of any assumptions or corrections, such as tests of normality and adjustment for multiple comparisons
☐	☒	A full description of the statistical parameters including central tendency (e.g. means) or other basic estimates (e.g. regression coefficient) AND variation (e.g. standard deviation) or associated estimates of uncertainty (e.g. confidence intervals)
☐	☒	For null hypothesis testing, the test statistic (e.g. *F*, *t*, *r*) with confidence intervals, effect sizes, degrees of freedom and *P* value noted<br>*Give P values as exact values whenever suitable.*
☒	☐	For Bayesian analysis, information on the choice of priors and Markov chain Monte Carlo settings
☒	☐	For hierarchical and complex designs, identification of the appropriate level for tests and full reporting of outcomes
☒	☐	Estimates of effect sizes (e.g. Cohen's *d*, Pearson's *r*), indicating how they were calculated

Our web collection on statistics for biologists contains articles on many of the points above.

Software and code

Policy information about availability of computer code

Data collection	No software was used for data collection
Data analysis	For single nuclear RNA sequencing (snRNAseq), demultiplexing, barcode processing, and gene expression quantifications were performed with the 10X Cell Ranger v3 pipeline using the GRCh38 (hg38). Doublets were identified and removed with DoubletDetection software (v2.4.0). Analyses involved the following R packages: Seurat (version 4.0.0) and Pagoda2 (version 1.0.2).<br>10X visium expression analysis, mapping, counting, and clustering was performed using Space Ranger (version 1.2.0, with the reference genome GRCh3-2020-A). Label transfer from SnRNAseq was done using Seurat (version 3.2.3). Differential expression analyses were done using R (v4.2.0) packages ReactomePA (v1.40.0) and ClusterProfiler (v4.4.4).<br>For imaging data, VTEA software version 1.0.3 , which is available for download through the FIJI plugin updater with the source code is available on GitHub, https://github.com/icbm-iupui/volumetric-tissue-exploration-analysis.

For manuscripts utilizing custom algorithms or software that are central to the research but not yet described in published literature, software must be made available to editors and reviewers. We strongly encourage code deposition in a community repository (e.g. GitHub). See the Nature Portfolio guidelines for submitting code & software for further information.

Data

Policy information about availability of data

All manuscripts must include a data availability statement. This statement should provide the following information, where applicable:

- Accession codes, unique identifiers, or web links for publicly available datasets
- A description of any restrictions on data availability
- For clinical datasets or third party data, please ensure that the statement adheres to our policy

All sequencing data were analyzed using the GRCh38 (hg38) reference genome. For published data from the KPMP/HuBMAP atlas, access to the raw snRNAseq data has been detailed in the Lake et al manuscript (Nature 2023, in press, https://doi.org/10.1038/s41586-023-05769-3). These raw sequencing data are under controlled access (human data) as they are potentially identifiable and can be accessed from the following respective sources: 1) KPMP data has been deposited at https://atlas.kpmp.org/repository/ and can be requested and made available by signing a data use agreement with KPMP; 2) the HuBMAP raw data are available for download from the database of Genotypes and Phenotypes; 3) processed data is available in the GEO Super-series: GSE183279 (https://www.ncbi.nlm.nih.gov/geo/query/acc.cgi?acc=GSE183279).

The new snRNAseq data generated for this study (raw sequencing and processed), along with all the Visium spatial transcriptomic data are available in GEO as the Super-series GSE231630 (https://www.ncbi.nlm.nih.gov/geo/query/acc.cgi?acc=GSE231630 or deposited in the HuBMAP portal (https://portal.hubmapconsortium.org/). A breakdown for the data availability of all papillary samples is presented in Supplementary Table 2.

Source data are provided with this paper .

For the imaging data: the source data for all the main and supplementary figures (3D imaging, CODEX and smFISH) are available at the following repository: doi: 10.5281/zenodo.7653239 (https://zenodo.org/record/7653239#.ZGIuGc7MKUk).

Human research participants

Policy information about studies involving human research participants and Sex and Gender in Research.

Reporting on sex and gender	Both sexes were included in this study since stone disease affects males and females. Sex was self identified. The sex of the participants is detailed in Supplemental Table 2 and 4.
Population characteristics	This study focused on patients with Calcium Oxalate stone disease and reference/ non-stone disease. The age of the participants in the tissue studies is provided in Supplemental table 2 as ranges. The characteristics of participants in the urine studies are provided in Supplemental table 4.
Recruitment	Described in methods. Biopsies were during surgical interventions for stone removal or from reference tissue as described. Urine studies as part of an ongoing study on stone formers and healthy participants.
Ethics oversight	We have complied with all ethical regulations related to this study. All experiments on human samples followed all relevant guidelines and regulations. The relevant oversight information is given based on tissue sources. Samples as part of the HuBMAP consortium were collected by the Kidney Translational Research Center (KTRC) under a protocol approved by the Washington University Institutional Review Board (IRB #201102312). Patients with stones disease who underwent biopsies were enrolled at Indiana University under IRB # 1010002261. Human reference nephrectomy papillary specimen were obtained from the Biopsy Biobank Cohort of Indiana under IRB #1906572234. Urine specimens from healthy participants and patients with a history of CaOx stones were obtained from an ongoing study at the University of Chicago under IRB protocol 09-164B.

Note that full information on the approval of the study protocol must also be provided in the manuscript.

Field-specific reporting

Please select the one below that is the best fit for your research. If you are not sure, read the appropriate sections before making your selection.

☒ Life sciences ☐ Behavioural & social sciences ☐ Ecological, evolutionary & environmental sciences

For a reference copy of the document with all sections, see nature.com/documents/nr-reporting-summary-flat.pdf

Life sciences study design

All studies must disclose on these points even when the disclosure is negative.

Sample size	Sample sizes were not predetermined by statistical methods due to nature of this study. Biopsy tissue specimens from human papilla are very rare, and the strength of the study lies in in the number of individuals analyzed, technologies represented for orthogonal validation and cells analyzed. Because of the rarity of the human tissue specimen from patient with stone disease, we used all samples available to us. Over 280,000 nuclei were included in the single nuclear atlas of which approximately 76,000 were derived from the papilla. Spatial transcriptomics (ST) experiments contributed over 10,000 papillary spots. This high number of nuclei provided the power to detect all major cell types and also novel cell subtypes including PapPC2 and undifferentiated cells. For ST, the 10000 papillary spots were sufficient to map every cell type in the papillary snRNAseq atlas. For the urine studies, the experiments were performed on all available samples, which were from participants consecutively recruited. The group sizes were sufficient to detect differences in the levels of MMP7 and MMP9.

Data exclusions	We excluded one sample from single cell RNAseq in the comparison between papillae from stone patient and reference tissue specimens because that specimen histologically had only partial papilla, and came from a donor who had severe kidney injury.
Replication	The complementary molecular assays and cross validation with different technologies strongly support our findings and could be considered equivalent to replication. All the experimental data is presented in the figures, supplementary information and source data. Replicates, when shown, are biological. For each specimen, we specified in Supplemental table 2, in which figure it was used.
Randomization	The assays were performed on all specimens available to us, and the results were obtained if the assays met quality control. No randomization was performed. This was not indicated for this analysis. Generation of data and processed files were agnostic to the disease conditions.
Blinding	All human specimens used in this study were de-identified, however select attributes (condition, age, sex) were available to all investigators. A majority of the analyses were not performed blind as these sample attributes were needed for accurate annotation of cell types and for the design of downstream analyses. The imaging performed (except the smFISH) was large scale on entire specimens and the analysis was global, independent of any sampling, and agnostic to the disease condition. The smFISH was performed for validation of cell types and there was no blinding indicated.

Reporting for specific materials, systems and methods

We require information from authors about some types of materials, experimental systems and methods used in many studies. Here, indicate whether each material, system or method listed is relevant to your study. If you are not sure if a list item applies to your research, read the appropriate section before selecting a response.

Materials & experimental systems

n/a	Involved in the study
☐	☒ Antibodies
☒	☐ Eukaryotic cell lines
☒	☐ Palaeontology and archaeology
☒	☐ Animals and other organisms
☒	☐ Clinical data
☒	☐ Dual use research of concern

Methods

n/a	Involved in the study
☒	☐ ChIP-seq
☒	☐ Flow cytometry
☒	☐ MRI-based neuroimaging

Antibodies

Antibodies used	The antibodies used in this study are described in details in the methods and also Supplementary Table 3. In the confocal 3D imaging experiments, the following primary antibodies were used for detection: anti-aquaporin 1 (Santa Cruz Biotechnology, Inc., Dallas, TX; sc-32737, dilution 1:50), anti-CD68 (Agilent Technologies, Santa Clara, CA; M087601, dilution 1:50), anti-phospho-c-JUN (Cell Signaling Technology, Danvers, MA; 9261, dilution 1:50), anti-aquaporin 2 (Life Technologies, Carlsbad, CA; PA522865, dilution 1:50) and anti-MMP7 (Santa Cruz Biotechnology, Dallas, TX; sc-515703, dilution 1:25). The Codex antibodies are listed in Supplementary table 3.
Validation	Validation of antibodies was performed before conjugation and after conjugation for CODEX, to make sure that the targets are still recognized (Supplementary Fig. 6). For most antibodies, validation was performed by confirming that the staining observed is consistent with vendor's data or published literature.<br>For the confocal 3D imaging, validation of antibodies and confidence in their staining is derived from several sets of data including vendors specifications, omitting primary antibody, well-established expected cell-type staining pattern for the indicated antibodies in the literature, referring to human protein atlas data where available and orthogonal validations in the multiomics data presented.<br>AQP-1: https://www.scbt.com/p/aqp1-antibody-1-22<br>CD68: https://www.agilent.com/store/en_US/Prod-M087601-2/M087601-2<br>p-c-JUN: https://www.cellsignal.com/products/primary-antibodies/phospho-c-jun-ser63-ii-antibody/9261- also this antibody validated in experimental models of injury (https://www.science.org/doi/abs/10.1126/scitranslmed.aaw3639)<br>AQP-2: https://www.thermofisher.com/antibody/product/Aquaporin-2-Antibody-Polyclonal/PA5-22865<br>MMP7: https://www.scbt.com/p/mmp-7-antibody-a-5
